# Risk of suicidal ideation, suicide attempts, and suicide deaths in persons with sleep apnea: Protocol for a systematic review and meta-analysis

**DOI:** 10.1371/journal.pone.0235379

**Published:** 2020-07-06

**Authors:** Yanxu Yang, Anna E. Ssentongo, Yunqi Pan, Matt Ciarletta, Vernon M. Chinchilli, Paddy Ssentongo

**Affiliations:** 1 Department of Public Health Sciences, Penn State Hershey College of Medicine and Milton S. Hershey Medical Center, Hershey, Pennsylvania, United States of America; 2 Department of Surgery, Penn State Hershey College of Medicine and Milton S. Hershey Medical Center, Hershey, Pennsylvania, United States of America; 3 Penn State Hershey College of Medicine and Milton S. Hershey Medical Center, Hershey, Pennsylvania, United States of America; 4 Center for Neural Engineering, Department of Engineering, Science and Mechanics, The Pennsylvania State University, State College, Pennsylvania, United States of America; Universitat Witten/Herdecke, GERMANY

## Abstract

**Aim:**

To estimate the pooled prevalence and incidence of suicidal ideation, attempts, and deaths in people with sleep apnea.

**Method:**

We will identify epidemiological studies reporting the prevalence or incidence rate of suicide in people with sleep apnea. We will search the following databases: PubMed (MEDLINE), Scopus, Cochrane Library, OVID (HEALTH STAR), OVID (MEDLINE) and Joana Briggs Institute EBF Database. No age, geographical location, study-design or language limits will be applied. This protocol was developed in accordance with the Preferred Reporting Items for Systematic Reviews and Meta-Analyses Protocols (PRISMA-P) guidelines. Two reviewers (YY and YP) will independently screen citations, abstracts and will identify full-text articles for inclusion, extract data, and appraise the quality and bias of included studies. Discrepancies will be resolved by consulting with a third researcher (MC). Study quality will be assessed by the Newcastle-Ottawa Scale. The primary outcomes will be the overall prevalence or incidence of suicidal ideation, attempts and completion and the risk of suicide in people with sleep apnea. For pooling of the studies, we will use a random-effects model with a logit transformation. The DerSimonian and Laird (DL) random-effects method will be used to estimate the pooled inter-study variance. We will assess the between-study heterogeneity using I^2^ statistics, and Cochrane’s Q statistic (significance level < 0.05). If the *I*^2^ is high (>75%), we will perform subgroup meta-analyses and conduct a meta-regression analysis to explore sources of study heterogeneity using study level median age, study-level proportions of race, gender, depression and quality scores. We will report effect estimates as suicide risk per 1000 individuals. Egger’s test and funnel plots will be used to assess publication bias, and adjusted estimates using trim and fill methods will be reported if publication bias is suspected.

**Ethics and dissemination:**

No ethics clearance is required as no primary data will be collected. The results of this systematic review and meta-analysis will be presented at scientific conferences and published in a peer-review journal. The results may shed more light on the burden of suicide risk among individuals with sleep apnea and may guide future population-specific interventions.

**Trial registration:**

PROSPERO registration number: CRD42020165404.

## Background

Sleep apnea is a potentially serious sleep disorder in which breathing is briefly and repeatedly interrupted during sleep [1]. Obstructive sleep apnea (OSA) is the most common type of sleep apnea, impacting on cardiovascular function. OSA has been associated with hypertension, coronary artery disease, cardiac arrhythmias, sudden cardiac death, and heart failure [2]. In the United States, it is estimated that 26% of adults aged 30–70 live with sleep apnea, and more than 25 million adults suffer from OSA [3]. According to the 2005–2014 National Survey among the United States male veterans, the prevalence of sleep apnea increased from 3.7% to 8.1% (p-value for trend <0.001) [4]. The MESA study conducted by Xiaoli Chen shows that the black population had higher risks of developing sleep apnea syndrome (adjusted odds ratio [OR] = 1.78, 95% confidence interval [CI]: 1.20, 2.63), compared with whites [4]. Furthermore, in a community-based study among a multiethnic Asian population, the prevalence of sleep apnea is 18.1%, and the prevalence in Malays is higher than that in China and India [5]. Although sleep apnea can occur at any age among all demographics, males, and people at young or middle age are more likely to develop sleep apnea [6, 7].

Suicide is a major public health problem and a leading cause of death in the United States. Suicidal ideation or attempts, as a possible outcome, is closely related to depression. Specifically, the rate of death caused by suicide among inpatients ever treated for depression is twice as high as outpatients [8]. Also, based on a new study from the Centers for Disease Control and Prevention, sleep apnea also is associated with probable major depression, regardless of factors like weight, age, sex or race [9]. A retrospective cohort study among 6,237 children shows that the risk of depressive disorders among children with sleep apnea was still significantly higher (hazard ratio [HR] = 2.25; 95% CI = 1.25–4.05). Moreover, boys with sleep apnea had a substantially higher risk of developing depression than those without sleep apnea (adjusted HR = 3.77; 95% CI, 1.82–7.54) [10]. However, few studies focused on the association between sleep apnea and suicide, and there is a gap in the literature in exploring the risk of suicide among people with sleep apnea.

To our knowledge, there is no systematic review and meta-analysis of the pooled prevalence or incidence of suicide in persons with sleep apnea. Thus, we plan to examine the prevalence or incidence of suicide and associated risk of suicidal ideation, attempts and completion. We hypothesize that suicide risk among individuals with sleep apnea is higher compared to the general population.

### Objectives

The objective of this study is to present a protocol for review and meta-analysis to ascertain the prevalence or incidence of suicidal ideation, attempt, and completion in sleep apnea and to delineate additional risk factors that may explain the high prevalence of suicide risk in individuals with sleep apnea. These may include race, gender, and depression.

Specific aims are:

- To examine the global prevalence or incidence of suicide ideation, attempt, and completion in persons with sleep apnea
- To delineate additional risk factors of suicide ideation, attempt and completion in people with sleep apnea.

### Review question

What is the prevalence or incidence of suicidal ideation, attempt, and deaths in individuals with sleep apnea?

## Method

We used the Preferred Reporting Items for Systematic Reviews and Meta-Analyses Protocols (PRISMA-P) 2015 statement and guidelines to inform the development of this protocol [11, 12]. See S1 Table for the checklist.

### Patient and public involvement statement

Patients were not involved in the development of this protocol.

### Study design

The inclusion criteria involve:

Studies that:

Reported suicide rates in sleep apnea, including prevalence and incidence
Published between January 1, 1960, to January 1, 2020
Published in any language.

We will exclude:

Studies not conducted in humans
Case reports, meeting abstracts, review papers, and commentaries

### Domain

We will include studies if they are related to sleep apnea and suicide.

### Population

We will include studies that report data generated from participants with sleep apnea, regardless of age, gender, and sex.

### Outcomes

The primary outcomes will be the overall rate of suicide deaths, suicide attempts, and suicidal ideation in individuals with sleep apnea. The secondary outcome will be other risk factors of suicide risk among people with sleep apnea, including age, gender, and major depression status.

### Search strategy

#### Geographical context

We will include studies from all over the world. However, regional differences will be estimated through subgroup analysis.

#### Database searches

The following databases will be searched: PubMed (MEDLINE), Scopus, Cochrane Library, OVID (HEALTH STAR), OVID (MEDLINE), and Joana Briggs Institute EBF Database. The snowballing method will be used to search the citation lists of included papers by using the ‘cited by’ tool in Google Scholar. We will make efforts to contact authors of ongoing studies and in-press literature for information regarding additional studies or missing data.

#### Search terms

Our keyword search will be based on Medical Subject Headings (MeSH) with various combinations of “Suicide*”, OR “Suicide attempt”, OR “Suicide Completion” AND “Sleep Apnea Syndromes *” OR“Sleep Apnea, Central” OR “Sleep Apnea, Obstructive”.

This search strategy will be further adapted and tailored for use with each database, using Boolean operators, truncations, proximity operators, and Medical Subject Heading, as appropriate for each database. For a complete list of search terms, see S1 Appendix.

#### Title and abstract screening

The citations will be imported into the Endnote software, and duplicate articles will be excluded. Two review team members (YY & YP) will independently screen studies in two stages. In the first stage, the two reviewers will independently screen titles and abstracts. They will document, with reasons, the studies excluded from the review [13].

#### Full-text screening and data extraction

In the second stage, two reviewers (YY & YP) will download/retrieve and assess independently full-text versions of selected abstracts. Data will be extracted from eligible papers identified during the abstract screening step. In the event of disagreement, the two authors (YY & YP) will discuss with each other and, if necessary, reviewers will ask a third author (MC) for her opinion to reach a consensus. When abstracts and subsequently included papers are not available in English, translators will be sought. We will extract the following information: first author, country in which the study was conducted, year of publication, study period, research methodology, total sample size, number of patients with suicidal ideation, number of patients with suicide attempt, number of patients with suicide completion, percent of study sample that was male, mean age, age with sleep apnea, number of patients with sleep apnea. In case of missing data, one attempt will be made to contact the corresponding authors of studies by email. If the author fails to provide additional information, a decision will be made as to whether to include the study in the final review [14].

#### Assessment of methodological quality of the papers

Two authors (YY and YP) will independently assess the quality of the papers included in the review. We anticipate finding cross-sectional, case-control, and cohort studies. Therefore, assessment of methodological quality will be conducted using the Newcastle-Ottawa Quality Assessment Scale, which is a validated tool for assessing quantitative cross-sectional, case-control, and cohort studies [15]. Studies will be included regardless of the risk of bias and quality scores, but sensitivity analysis will be conducted to ascertain the impact of their inclusion.

### Data synthesis and analysis

We will use the metaprop function of the meta-package in R Statistical Software for analysis [16]. We will use the random-effects model with a logit transformation of proportions for the pooling of studies [17]. The confidence intervals will be calculated using the exact binomial (Clopper-Pearson) interval method. The approximate variance of a logit transformed proportion can become infinite if the number of events is zero or equal to the sample size. As extensively discussed by Schwarzer et al. 2019,[18] a small increment will be added to each denominator to yield a finite variance estimate. The DerSimonian and Laird (DL) random-effects method will be used to estimate the pooled inter-study variance [19]. Even if it is determined that the between-study heterogeneity is low, the random-effects model still will be applied [20]. We will do the five separate pooled analyses for prevalence or incidence rate separately. They will include the prevalence of 1) suicidal ideations, 2) suicide attempts, the incidence of 3) suicidal ideations, 4) suicide attempts and, 5) suicidal deaths in individuals with OSA. We will graphically display individual and pooled estimates with forest plots. Inter-study heterogeneity will be assessed using *I*^2^ statistics, expressed as % (low (25%), moderate (50%), and high (75%) and Cochrane’s *Q* statistic (significance level < 0.05) [21, 22]. We will use subgroup meta-analyses to perform sensitivity analysis and investigate geographical differences in the risk of suicide. We will conduct a meta-regression analysis, using study level median age, and study level gender proportions, year of study, the proportion of study population with sleep apnea, and risk of suicide in persons with sleep apnea [23]. We will report the incidence rate of suicide or prevalence of suicidal ideation, attempts, or completion per 1000 population.

#### Risk of bias in individual studies

Egger’s test and funnel plots will be used to assess publication bias. In the presence of asymmetrical funnel plots and significant Egger’s test, trim and fill analyses will be conducted, and adjusted effect sizes will be reported. In addition, influence analysis will be performed. The analysis excludes and replaces one study at a time (leave-one-out method) from the meta-analysis and calculating the pooled effect size for the remaining studies.[24, 25] A second sensitivity analysis will be performed by subgroup analysis between high quality and medium/low-quality studies.

### Presentation of results and reporting

The PRISMA guidelines will be used, and the checklist will accompany the publication. Quantitative data will be summarized and presented in tables, forest plots, and charts. The prevalence and incidence of suicide in sleep apnea will be presented by continents and by study design.

### Potential amendments

The review of the protocol commenced in 2020, and the study is expected to be completed by 2021. We do not foresee amendments to this protocol. However, in case a need for modification should arise, it will be registered and reported in this journal.

### Patient and public involvement

Patients were not involved in the development of this systematic review protocol.

## Conclusion

Ours will be the first comprehensive systematic review and meta-analysis to synthesize the current literature on the risk of suicide in persons with sleep apnea. This study will contribute to assessing the high-risk population of suicide and proposing an intervention plan.

## Dissemination

The results of this systematic review and meta-analysis will be presented at conferences and published in a peer-review journal. The results will guide future population-specific interventions.

## Supporting information

S1 Table PRISMA-P checklist. (DOC)

S1 Appendix MeSH terms. (DOCX)
