# Supplementary material for: Risk of suicidal ideation, suicide attempts, and suicide deaths in persons with sleep apnea: Protocol for a systematic review and meta-analysis
Source: PLoS One. 2020 Jul 6;15(7):e0235379. doi: 10.1371/journal.pone.0235379 (PMC7337338; doi:10.1371/journal.pone.0235379)
Supplement: S1 Appendix — (DOCX) [file pone.0235379.s002.docx]

Search Terms:

1. (("Suicide"[Mesh] OR "Suicide, Attempted"[Mesh] OR "Suicide, Completed"[Mesh])) AND ("Sleep Apnea Syndromes"[Mesh] OR "Sleep Apnea, Central"[Mesh] OR "Sleep Apnea, Obstructive"[Mesh])

2. "Suicide"[Mesh] OR "Suicide, Attempted"[Mesh] OR "Suicide, Completed"[Mesh]

3. "Sleep Apnea Syndromes"[Mesh] OR "Sleep Apnea, Central"[Mesh] OR "Sleep Apnea, Obstructive"[Mesh]
